# Retinoic Acid Signaling Organizes Endodermal Organ Specification along the Entire Antero-Posterior Axis

**DOI:** 10.1371/journal.pone.0005845

**Published:** 2009-06-10

**Authors:** Elke Bayha, Mette C. Jørgensen, Palle Serup, Anne Grapin-Botton

**Affiliations:** 1 Swiss Institute for Experimental Cancer Research, Ecole Polytechnique Fédérale de Lausanne, Lausanne, Switzerland; 2 Department of Developmental Biology, Hagedorn Research Institute, Gentofte, Denmark; Harvard University, United States of America

## Abstract

**Background:**

Endoderm organ primordia become specified between gastrulation and gut tube folding in Amniotes. Although the requirement for RA signaling for the development of a few individual endoderm organs has been established a systematic assessment of its activity along the entire antero-posterior axis has not been performed in this germ layer.

**Methodology/Principal Findings:**

RA is synthesized from gastrulation to somitogenesis in the mesoderm that is close to the developing gut tube. In the branchial arch region specific levels of RA signaling control organ boundaries. The most anterior endoderm forming the thyroid gland is specified in the absence of RA signaling. Increasing RA in anterior branchial arches results in thyroid primordium repression and the induction of more posterior markers such as branchial arch *Hox* genes. Conversely reducing RA signaling shifts *Hox* genes posteriorly in endoderm. These results imply that RA acts as a caudalizing factor in a graded manner in pharyngeal endoderm. Posterior foregut and midgut organ primordia also require RA, but exposing endoderm to additional RA is not sufficient to expand these primordia anteriorly. We show that in chick, in contrast to non-Amniotes, RA signaling is not only necessary during gastrulation, but also throughout gut tube folding during somitogenesis. Our results show that the induction of *CdxA*, a midgut marker, and pancreas induction require direct RA signaling in endoderm. Moreover, communication between *CdxA* ^+^ cells is necessary to maintain *CdxA* expression, therefore synchronizing the cells of the midgut primordium. We further show that the RA pathway acts synergistically with FGF4 in endoderm patterning rather than mediating FGF4 activity.

**Conclusions/Significance:**

Our work establishes that retinoic acid (RA) signaling coordinates the position of different endoderm organs along the antero-posterior axis in chick embryos and could serve as a basis for the differentiation of specific endodermal organs from ES cells.

## Introduction

Endodermal cells contribute to different organs along the antero-posterior (AP) axis, including the pharynx, esophagus, stomach, duodenum, small and large intestine. Furthermore, associated organs arise along this axis from head to tail: thyroid, thymus, parathyroid, lungs, liver, pancreas and caecum. Much progress has been made in the identification of transcription factors that govern endodermal organ differentiation. Previous work has demonstrated that several of these organ primordia are initially induced by signals from neighboring mesoderm. Accordingly, ectopically transplanted endoderm can be re-specified by mesoderm that comes from a different AP location [1]–[3]. Some of the signals sent by the mesoderm to the endoderm have been molecularly characterized but in most cases the signals identified do not induce a specific organ. For instance, FGF10 signaling is required in the emerging primordia of the thyroid, thymus, lungs, pancreas and caecum [4]–[9]. BMP4 is required for at least the thymus and liver anlage [10], [11]. A one to one scenario in which a specific mesodermal signal induces a given organ at a specific position is therefore unlikely. In contrast, there is emerging evidence that different signaling thresholds of a few extracellular signals induce different organs. Wnts, FGFs and retinoic acid (RA) are soluble signaling factors that posteriorize the neurectoderm by acting in a graded manner. There is evidence that they have similar activities in endoderm [1], [3]. We and others have recently shown that in chick and mouse, endoderm that has not received FGF4 has anterior foregut characters and more posterior endoderm is progressively induced by increasing exposure to FGF4 [2], [12]. Similarly, in *Xenopus laevis*, Wnt/beta-catenin activity must be repressed in the anterior endoderm to maintain foregut identity whereas high beta-catenin activity in the posterior endoderm inhibits foregut fate while promoting intestinal development [13]. It is not yet clear whether this activity is graded. In this study, we have investigated the role of the RA signaling pathway on chick endoderm regionalization along the AP axis between late gastrulation and early somitogenesis.

RA is the biologically active derivative of vitamin A, which is oxidized in a two step process. It activates gene expression via direct binding to different nuclear receptors that are expressed as various isoforms during embryogenesis. The signaling activity is limited by further oxidation through the Cyp26 enzymes of the cytochrome p450 family. The role of RA in endoderm has been previously addressed in different studies. RA signaling is required for branchial arch morphogenesis in mice [14]–[16]. RA is also necessary for pancreas formation in *Xenopus laevis*, zebrafish and mice [17]–[24]. In this article, we use the chick model system to provide a global view on how RA influences organ position at different levels along the AP axis. Our results show that the most anterior foregut can only form in the absence of RA. Exposure to exogenous RA inhibits genes normally expressed in the most anterior endoderm, while genes transcribed at the level of branchial arches are activated and expanded anteriorly. Concomitantly, inhibiting RA signaling at the level of its receptors decreases branchial arch marker expression and shifts them posteriorly. We show that genes expressed posterior to the first somite level, which define cells that give rise to the pancreas and small intestine, absolutely require RA but are not shifted anteriorly by exogenous RA. In contrast to previous observations in zebrafish, we show that expression of the midgut marker *CdxA* requires RA and that this signaling is directly occurring in endoderm. Our results also show that in contrast to *Xenopus laevis* and zebrafish, endoderm patterning by RA extends beyond gastrulation to the stages of gut tube folding. This difference may ensure that cells of the dorsal and ventral gut tube, which originate from different positions along the AP axis, eventually have identical AP identities [25], [26]. Furthermore, we demonstrate that RA and FGF4 synergistically pattern definitive endoderm.

## Materials and Methods

### Chick embryo isolation and culture

Fertilized White Leghorn chicken eggs (E. Pavillard, Orny, Switzerland) were incubated at 38°C to obtain stage HH 3–4, stage HH 8, or stage HH 11 embryos [27]. Chick embryos were isolated and placed in a modified New culture for *in vitro* manipulation [28]. Briefly, eggs were opened into a 10 cm culture dish, and the albumen was scraped off from the embryonic region with a razor blade. A 20 by 20 mm piece of Watman paper #1 (Schleicher & Schuell) with a 5 mm hole in the center was placed over the embryo, and the embryo was cut away and placed ventral (endoderm) side up on a plate containing 0.8% Bacto agar (AxonLab/Applichem), 50% albumin and 0.3% glucose (Serva) in saline. All animal experiments were performed in agreement with the regulations of the Swiss veterinary office of the canton of Vaud.

### Application of RA

*All trans* RA was purchased from Sigma. For the bead grafting approach, AG1-X2 anion exchange resin (BioRad) containing chloride bound beads ranging from 200–400 µm in diameter were equilibrated in formate for which they have a low affinity. Formate was then replaced by incubating them over night at 4°C in a solution of ethanol with 0, 10^−3^ M or 10^−4^ M RA and subsequently washed with PBS. For gastrula stage engraftment, beads were placed on endoderm at locations determined from Kimura et al. [29]. For somatic stage engraftment, beads were grafted at different levels along the AP axis either on the lateral plate endoderm immediately adjacent to somites or on the lateral side of the already closed foregut endoderm. As an alternative to bead-loaded RA, RA was included directly in the New culture medium at 10^−6^ M or 10^−7^ M. Control plates were prepared by adding the corresponding amount of ethanol only. This allowed the use of lower RA concentrations for an identical effect but did not allow limiting exposure to a small area.

### Application of AGN193109

The RA antagonist AGN193109 was synthesized at Novo Nordisk A/S (DK) [30]. Its activity was tested in RA-responsive P19 embryonic carcinoma cells using *RARβ*, a direct target of RA signaling, as a readout [31], [32] (Fig. S1). For the treatment of chick embryos, a 10^−2^ M AGN193109 stock solution in DMSO was prepared and 10^−5^ M AGN193109 or pure DMSO as control was included in the culture medium. The embryos were incubated and placed as described above.

### Application of FGF4

Recombinant FGF4 was purchased from R&D Systems Inc. Heparin acrylic beads (Sigma) ranging from 150–250 µm in diameter were soaked in a solution of PBS alone or with 1 mg/ml FGF4. For gastrula stage engraftments, beads were cut in half and placed flat side down on endoderm being careful not to tear the endoderm.

### Application of SU5402

SU5402 was purchased from Calbiochem. The FGF inhibitor was directly included in the New culture medium by adding SU5402 (50 mM stock solution in DMSO) to a final concentration of 20 µM or pure DMSO as control.

### In ovo electroporation

Electroporation was performed on embryos between the 18- and 22-somite stage (stage 13–14 HH), corresponding to about 54 h of incubation at 38°C, as previously described [33]. After electroporation, eggs were resealed with tape and placed at 38°C for 28–30 h or 48 h. Dominant-active (VP16 fusion) and -negative (truncation at amino acid 403 of hRAR) retinoic acid receptors cloned into pCIG were provided by S. Sockanathan and T. Jessell. The vectors lead to GFP co-expression [34]–[36].

### *In situ* hybridization and sectioning of chick embryos

Whole mount *in situ* hybridization was performed as described previously [37]. Briefly, embryos were fixed in 4% paraformaldehyde, dehydrated in methanol, rehydrated, treated with proteinase K (10 µg/ml) for 30 seconds up to 3 minutes depending on their stage, and postfixed in 4% paraformaldehyde and 4% glutaraldehyde. Embryos were hybridized over night at 70° C in hybridization buffer (50% formamide, 1.3× SSC, 5 mM EDTA, 50 µg/ml Yeast RNA, 0.2% Tween 20, 0.5% CHAPS, 50 mg/ml Heparin), containing 1 µg/ml RNA probe. Embryos were washed and incubated over night with an anti-Digoxigenin antibody (Roche, 1∶2000). Staining was developed with 4.5 µl/ml of NBT stock solution (75 mg/ml) and 7 µl/ml of BCIP stock solution (25 mg/ml). *In situ* hybridization probes were previously published: *RARα, β* and *γ* [38], *HoxB4* [39], *Prox1* [40], *Pdx1* [41], *CdxA* [42], *Hex1* [43], *Nkx2.1* [44], *Nkx6.2* [45], *γ-fibrinogen* [46], *HoxA2* [47], the *Cyp26A1* and *Raldh2* probes were generated by RT-PCR amplification from stage HH 20 chick cDNA. The primers used to amplify the cDNA were: *Raldh2* (antisense: 5′-gctcttctgcactatgtgg-3′; sense: 5′-atggcatctctgcatctgc-3′); *Cyp26A1* (antisense: 5′-tcagatttggccgctgaaacc-3′; sense: 5′- atgggcttctccgctctggtcg-3′). The cDNAs were cloned into pGEM-Teasy (Promega). For sections, whole mount in situ stained embryos were embedded in 15% sucrose-gelatine and 15 µm cryo-sections were collected.

### Immunocytochemistry

Whole mount triple immunostainings of chicken embryos were performed as described [48] using the following primary antibodies: mouse anti-Nkx6.1 (F55A10; Developmental Studies Hybridoma Bank/BCBC, 1∶1000), goat anti-Pdx1 (kind gift from Chris Wright, 1∶2000), guinea pig anti-glucagon (4031-01F; Linco, 1∶10000), rabbit anti-GFP (8367-1; Clontech, 1∶2000). Secondary antibodies: Cy2-conjugated donkey anti-goat and anti-rabbit, Cy3-conjugated donkey anti-guinea pig and anti-mouse, Cy5-conjugated donkey anti-guinea pig and anti-mouse (all from Jackson ImmunoResearch Laboratories, 1∶500)

Whole mount immunocytochemistry with rabbit anti-GFP antibody (Invitrogen, 1∶1000) was used to reveal GFP-expressing cells after in situ hybridization and was developed either with goat anti-rabbit horseradish peroxydase (Jackson Immunologicals Research) and diaminobenzidine or donkey anti-rabbit Alexa488 (Molecular Probes).

## Results

### Endogenous RA production, reception and degradation in the vicinity of the endoderm

**Figure 1:**
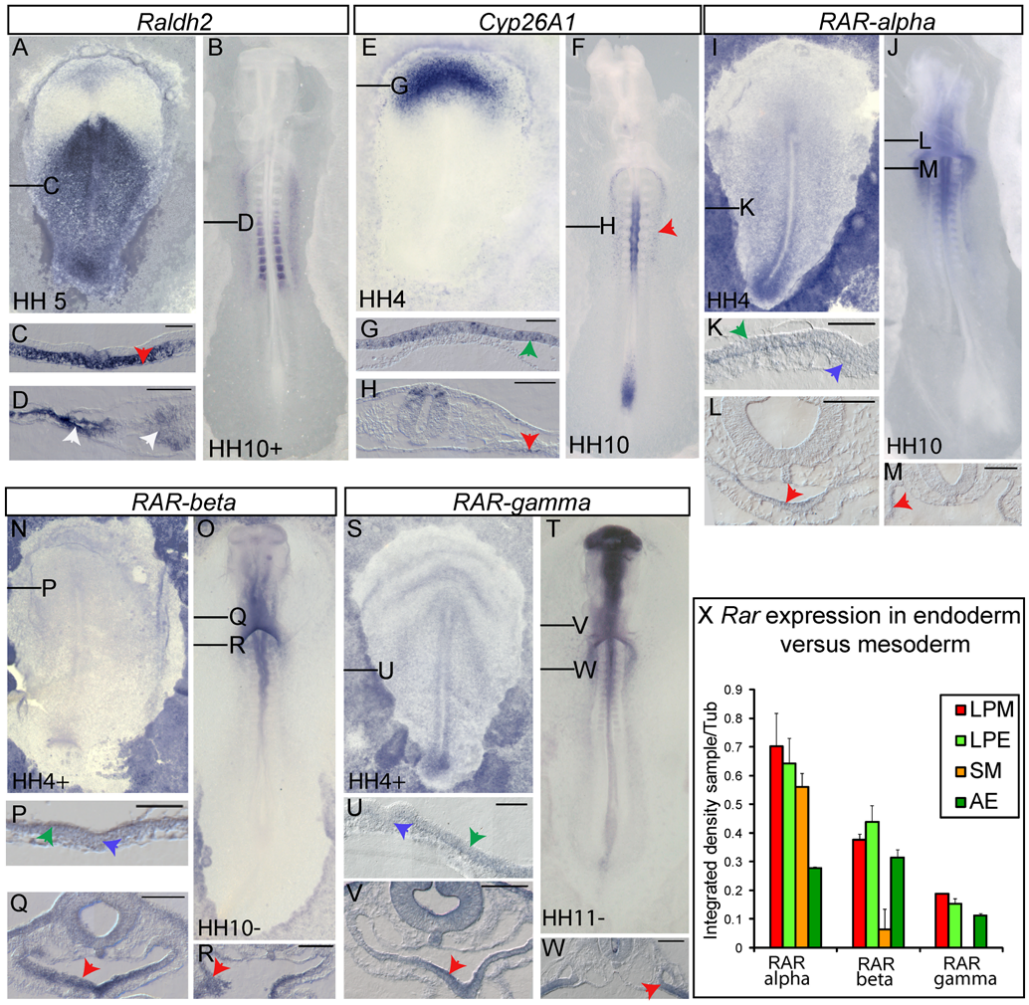
Endogenous RA signaling can be activated in endoderm. Whole mount *in situ* hybridization analysis of *Raldh2* (A–D), *Cyp26A1* (E–H), *RARα* (I–M), *RARβ* (N–R) and *RARγ* (S–W) at gastrulation or early somitogenesis. Exact stages are indicated on whole mount pictures, ventral views, anterior to the top. 15 µm cryo-sections are shown in (C,D,G,H,K–M,P–R,U–W), dorsal side to the top. Red arrowheads point to expression in endoderm, blue arrowheads indicate cells within the PS, green arrowheads show the epiblast andwhite arrowheads point to *Raldh2* expression in somites and LPM. Black lines in the whole mounts indicate the relative plane of sections. Scale bars are 100 µM. (X) RT-PCR analysis of *RAR* expression in endoderm versus mesoderm, which were harvested at stage HH 10. The integrated density of resulting bands were measured and normalized to *tubulin* expression. Each sample was made in duplets and bars show the mean. Error bars indicate standard deviation. AE, axial endoderm; LPE, lateral plate endoderm; LPM, lateral plate mesoderm; SM, somitic mesoderm.

To establish whether there is a source of retinoids in endoderm or in neighboring tissues, we investigated the expression of RA-synthesizing enzyme *Raldh2*. At stage HH 4/5, *Raldh2* is expressed rostral to the node in the hypoblast and definitive endoderm. Posterior to the node it is present in the mesendoderm and weaker in the primitive streak (Fig. 1A, C). During early somitogenesis (HH 10/11) *Raldh2* is detected in lateral plate mesoderm (LPM) and somitic mesoderm (SM) (Fig. 1B, D).

A cell will respond to RA only when it expresses the *RAR* receptors. In chick, three *RAR* genes have been identified and for two of those, *RARα* and *RARγ*, two different isoforms have been reported [49], [50]. The probes used to detect *RAR* transcripts by whole mount *in situ* hybridization do not distinguish between different isoforms. Around HH 4+, *RARα* and *RARγ* are ubiquitously expressed with highest levels around the posterior primitive streak (Fig. 1I, S). *RARβ* transcripts were detected equally in the entire epiblast (Fig. 1N). Sections show that all *RAR*s are present in the epiblast and cells within the primitive streak show weak expression (Fig. 1K, P, U). The latter expression sites include cells which are fated to become endoderm [29], [51], [52]. We could not clearly establish whether RARs are expressed in definitive endoderm during gastrulation because expression is weak and the endodermal layer is thin. At HH 10, all *RAR*s were anteriorly expressed in the neural tube and the ventral foregut endoderm with highest levels for *RARβ* (Fig. 1L, Q, V). Posterior to the anterior intestinal portal (AIP), we detected all *RAR*s in lateral plate endoderm and in the neural tube with highest levels for *RARβ* and *RARγ* (Fig. 1M, R, W).

At stage HH 10, the axial and the posterior endoderm are thin layers that have not yet thickened. This makes it difficult to detect staining especially in this area. Therefore, we assayed dissected lateral plate mesoderm (LPM), lateral plate endoderm (LPE), axial endoderm (AE) and somitic mesoderm (SM) of stage 10 chick embryos (levels somite 3–10) for *RAR* expression by semi-quantitative RT-PCR. α-*Tubulin* was used for normalization. We found *RAR*s to be expressed in endoderm and mesoderm, even medially, although *RARβ* and *RARγ* were not expressed or only slightly in somites (see columns SM in Fig. 1X).

RA signaling is spatially and temporally restricted by *Cyp26A1* activity which metabolizes RA to an inactive form. During gastrulation *Cyp26A1* is expressed anterior to the node in the epiblast layer (Fig. 1E, G). Around stage HH 4+ there is an additional small expression domain around the node which was found to be localized in the mesoderm below [53]. At stage HH 10, we detected *Cyp26A1* expression in the dorsal half of the medial spinal cord and posterior to the AIP in LPE and in the tail bud (Fig. 1F, H). *Cyp26A1* is a direct target of RA signaling [54] and its expression in the LPE provides, therefore, a readout of transcriptional activation by RA. However, it is likely that the *Cyp26A1* gene is also subject to RA-independent tissue-specific regulation and RA signaling can be active beyond *Cyp26A1* expression domains [55].

In summary, these expression data show that RA is synthesized in endoderm at stage HH 5 and in mesoderm closely associated to the endoderm at stage HH 10, where it may induce RAR-mediated transcription to pattern the future gut tube.

### Exogenously applied RA activates direct target genes within endoderm

**Figure 2:**
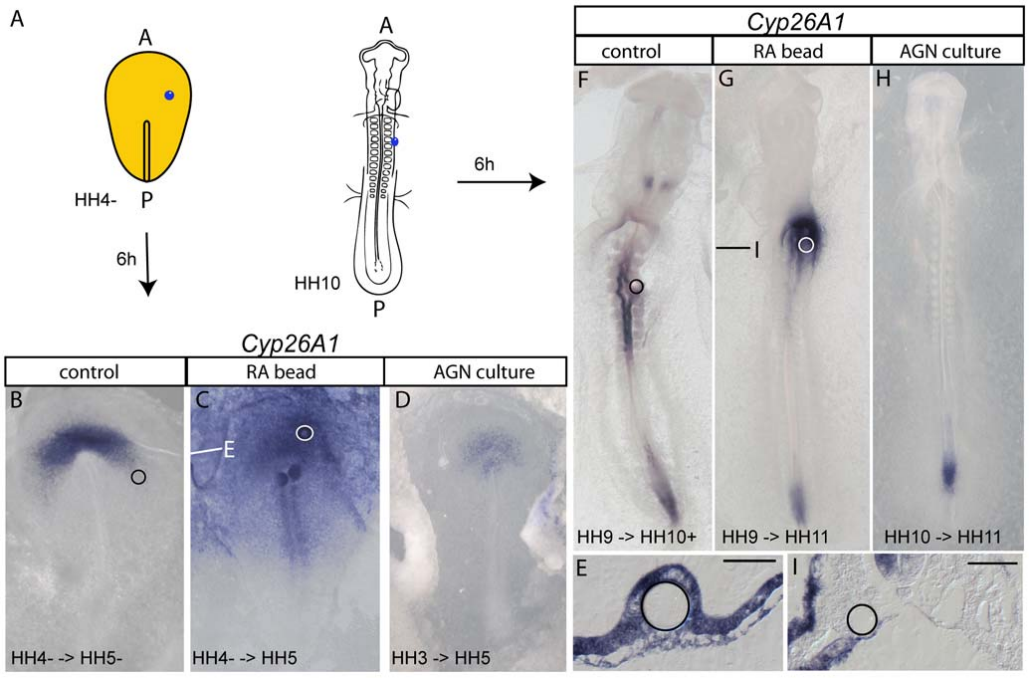
Exogenous RA activates ectopic gene transcription in the endoderm. (A) is a schematic illustration showing the initial grafting position of the RA bead in stage HH 4^−^ or HH 10 chick embryos. (B–I) show whole mount *in situ* hybridization analysis of *Cyp26A1* in control (B,F), RA bead grafted (C,G) or inhibitor treated embryos (D,H), ventral view, anterior to the top. Beads were soaked in ethanol (control) or ethanol containing 10^−3^ M RA and were grafted onto the endoderm and analyzed 6 hours later (approximately HH 5 or HH 11, respectively). For inhibition, embryos were treated with 10^−5^ M AGN193109 added to the culture medium at HH 3^+^ or HH 10, incubated 6 hours and then analyzed (approximately HH 4–5 or HH 11, respectively). Exact stage of grafting and analysis are given in each picture. Position of beads is marked by a circle and lines give the plane of sections shown in (E and I), ventral side down. Scale bars are 100 µM. Note broad induction in (E) and unilateral induction ventrally in (I). A, anterior; h, hours; P, posterior.

To investigate whether endoderm responds to exogenous RA, we made use of the direct RA target gene *Cyp26A1.* Either at stage HH 4 (late primitive streak stage) or at stage HH 10 (10 somite stage), we grafted beads soaked in RA (10^−3^ M or 10^−4^ M) onto the endoderm in modified New cultures (Fig. 2A) [28]. After 6 hours of incubation, these embryos were assayed for the expression of *Cyp26A1*. We chose this relatively short incubation period to reduce the possibility of indirect target gene induction via RA activation in neighboring cells, notably in mesoderm. At HH 5, embryos exposed to RA-loaded beads showed broad *Cyp26A1* induction around the bead in endoderm and in the epiblast (Table 1; Fig. 2C, E) as well as in endoderm and surface ectoderm at HH 11 (Table 1; Fig. 2G, I). The range of *Cyp26A1* induction around a bead soaked with 10^−3^ M RA can be estimated to be half of the embryo during gastrulation and to diffuse to the length of 3 somites during somitogenesis. Lower concentrations (10^−4^ M) of RA induced *Cyp26A1* mRNA in a shorter range (not shown). Likewise, either 10^−6^ M and 10^−7^ M RA included into the medium either at gastrula or somitic stage resulted in elevated *Cyp26A1* expression in its endogenous domains. However, induction in ectopic areas was not observed.

**Table 1:** Effects of RA on endoderm marker gene expression.

Application	[RA]	Cyp26A1	HoxA2	HoxB4	Hex	Nkx2.1	Prox1	γ-fibrinogen	Pdx1	CdxA
Graft at gastrulastage (HH 4)										
Bead graft	10^−3^ M	6/6	ND	1/1	7/7	6/6	ND	ND	0/5	0/2
	10^−4^ M	4/4	ND	8/8	5/6	5/5	ND	ND	0/8	0/1
	control	2	ND	4	9	12	ND	ND	6	2
Culture med	10^−6^ M	2/4	ND	0/4	3/3	2/2	ND	ND	0/5	ND
	10^−7^ M	0/3	ND	0/5	3/3	1/1	ND	ND	0/4	ND
	control	2	ND	3	3	1	ND	ND	6	ND
Graft at somite stage (HH 10)										
Bead graft	10^−3^ M	8/8	6/6	17/17	5/5	2/2	6/6	0/3	0/18	0/15
	10^−4^ M	7/7	6/6	5/5	5/5	3/3	ND	ND	0/21	0/8
	control	10	4	16	4	3	3	2	13	8
Culture med	10^−5^ M	1/1	ND	ND	5/5	ND	ND	ND	0/5	ND
	10^−6^ M	4/7	ND	3/4	2/6	ND	ND	ND	0/21	0/3
	10^−7^ M	0/6	ND	0/4	3/8	ND	ND	ND	0/23	0/3
	control	8	10	5	14	ND	ND	3	26	3

Stage indicates the stage of treatment; Bead graft, grafting of RA-soaked beads; Culture med, RA was applied to the culture medium; ND, not done;

Phenotypes: *Cyp26A1*, concentration-dependent induction around the bead; *HoxB4* and *HoxA2*, anterior expansion of the domain & increased levels of expression; *Hex*, comprise embryos collected at HH 5–6 and HH 14; at HH 5–6, *Hex* is partially repressed and at HH 14, *Hex* is inhibited in thyroid domain, liver domain remains unaffected; *Nkx2.1*, inhibited thyroid expression; *Prox1*, repression close to the bead. *γ-fibrinogen, Pdx1* and *CdxA*, no effect.

In order to block RA signaling, we used AGN193109 (10^−6^ M) in the culture medium to inhibit activity of all *RAR*s [30]; Supplementary Figure 1). Inhibition of RA signaling resulted in diminished *Cyp26A1* expression at gastrulation stage (Fig. 2D; Table 2) and complete absence of *Cyp26A1* transcripts in the trunk of stage HH 11 embryos (Fig. 2H; Table 2). Expression in the tail bud is independent of RA signaling at this stage. These experiments prove that *Cyp26* expression in endoderm is indeed RA-dependent and also demonstrate an active endogenous RA pathway in endoderm at gastrulation and somitogenesis.

**Table 2:** Effects AGN193109 on endoderm marker gene expression.

Stage	[AGN]	Cyp26A1	HoxA2	HoxB4	Hex	Nkx2.1	Prox1	γ-fibrinogen	Pdx1	CdxA
Early gast (HH 3+)	10^−5^ M	5/5	0/5	ND	7/7	0/5	0/4	4/4	2/2	2/3^1^; 0/3^2^
Gastrula (HH 4)	10^−5^ M	6/8	ND	3/3	0/4	0/6	0/1	ND	4/4	1/2^1^; 1/2^2^
Early som (HH 7)	10^−5^ M	ND	ND	ND	ND	ND	ND	ND	5/5	2/3^1^; 1/3^2^
Som (HH 10)	10^−5^ M	5/5	ND	1/3	0/3	0/4	ND	ND	0/3	0/3^1^; 1/3^2^
	control	11	12	3	10	13	6	5	8	8

Inhibitor was applied to the culture medium; Stage indicates the stage of treatment; ND, not done;

Phenotypes: *Cyp26A1*, expression is inhibited in discrete areas (see text); *HoxB4*, posterior shift of expression domain and decreased expression level; *Hex*, comprise embryos collected at HH 5–6 and HH 14; at HH 5–6, *Hex* is reduced and at HH 14, *Hex* is ectopically expressed between thyroid & liver; *Nkx2.1*, *HoxA2* and *Prox1* no change in expression pattern. *γ−fibrinogen*, slight increase in expression. *Pdx1*, complete repression; *CdxA*, two phenotypes^1^ partial or complete repression or^2^ anterior expression border is posteriorly shifted.

### RA patterns branchial arch endoderm in a graded manner

To investigate how altered RA signaling affect subsequent gut tube patterning, we either grafted RA-soaked beads (10^−3^ M or 10^−4^ M) onto chick embryos or included different concentrations of RA (10^−6^ M or 10^−7^ M) in the culture medium. In these embryos a set of transcription factors expressed in anterior foregut endoderm were then analyzed. Grafting beads limits the area exposed to RA and thereby teratogenic effects. At somite stages it conserves the contro-lateral side as an internal control. Applying RA into the culture medium guaranties a more even exposure to more physiological levels of RA. However, both approaches showed similar effects on marker expression.

**Figure 3:**
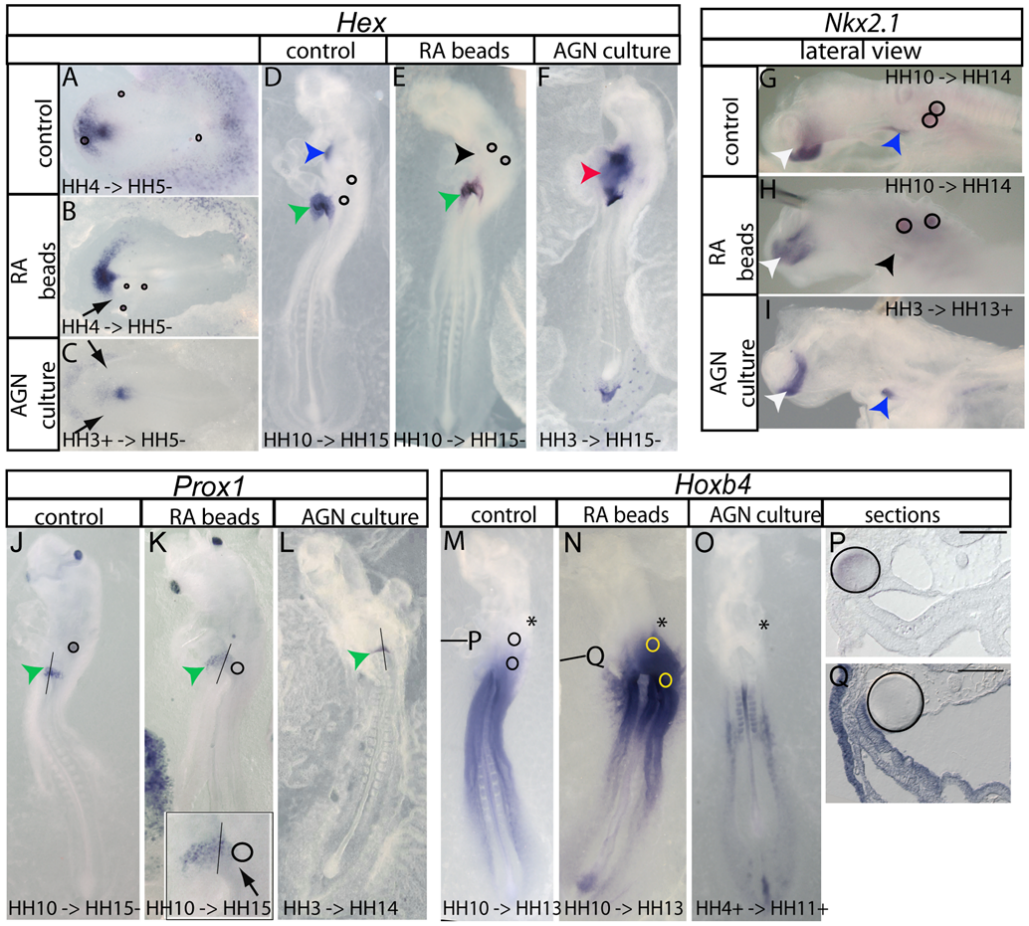
RA patterns pharyngeal endoderm in graded manner. RA beads (10^−3^ M) and control beads were grafted at HH 10 onto endoderm and embryos were analyzed at different stages corresponding to the time when the specific marker is expressed (D,E,G,H,J,K,M,N). *Hex* expression was also analyzed in embryos grafted at HH 4 and analyzed at HH 5 (A,B). Inhibition of RA was conducted in stage HH 3^+^ or HH 4 chick embryos by adding 10^−5^ M AGN193109 to the culture medium (C,F,I,L,O). Exact stages of treatment and analysis are indicated in each picture. Whole mount *in situ* hybridization analyzed embryos for *Hex* (A–F), *Nkx2.1* (G–I), *Prox1* (J–L) and *HoxB4* (M–O). In lateral views of *Nkx2.1* (G–I), surface ectoderm was removed to see better the staining. Box in (K) shows higher magnification of *Prox1* inhibition by RA treatment. Although liver *Hex* expression may appear reduced in E, this is not reproducibly observed. The black line marks the midline at the AIP. *HoxB4* is expressed in all three germ layers, therefore, induction in endoderm has been verified by 15 µm sections (P,Q) marked by black lines in the corresponding whole mount embryos. Anterior is always at the top except in (G–I) where anterior is shown to the left. Scale bars are 100 µM. Asterisks mark the otic vesicle. Black arrowheads mark the loss of thyroid gene expression in RA treated embryos. Black arrows point to loss of *Hex* or *Prox1* expression. Blue arrowheads indicate normal thyroid expression of *Hex* or *Nkx2.1*. Green arrowheads point to the liver bud. Red arrowhead shows ectopic *Hex* expression. White arrowhead marks forebrain expression of *Nkx2.1*. Circles mark the position of grafted beads.

*Hex* is the most anterior and earliest endoderm marker. We analyzed *Hex* expression in embryos grafted at stage HH 4- and collected them at stage HH 5–6. At this early stage, *Hex* marks the presumptive foregut endoderm, which gives rise to organs including the liver, thyroid and ventral pancreas [56]. Upon activated RA signaling, *Hex* expression is reduced (Fig. 3A, B; Table 1) either generally when RA is provided in the culture medium or unilaterally around the bead when it is locally delivered. This suggests that anterior endoderm fate is repressed by *all-trans* RA. To examine the later consequences of this early exposure to RA, we fixed the embryos at stage HH 15 when *Hex* expression defines thyroid and liver primordia [56]. RA treatment of chick embryos specifically inhibits *Hex* expression in the thyroid gland but not in the liver (Fig. 3D, E; Table 1). At stage HH 11, *Nkx2.1* gene activity defines the thyroid in ventral foregut endoderm and the hypophysis in the forebrain [57]. In RA-treated embryos at stage HH 14, analysis of *Nkx2.1* confirms that thyroid-specific genes are inhibited through exogenous RA (Fig. 3G, H; Table 1) suggesting that thyroid primordia cells are affected. Similar results were obtained when embryos are exposed to RA at stage HH 10 and fixed at HH 14 suggesting that RA can block anterior-most endoderm identity until somitogenesis (Table 1). To ascertain further the effect of RA on liver induction, we investigated two independent liver markers after RA exposure at stage HH 10. *Prox1*, which is specifically expressed in the differentiating liver bud at HH 13/14 [46], is locally reduced when RA signaling is ectopically activated (Fig. 3M, N). However we could see no effect on the independent marker γ-fibrinogen (Fig. S3). These findings suggest that RA treatment does not impair liver positioning along the main axis of the gut and does not generally inhibit later liver differentiation, although it may affect specific differentiation markers.

To investigate whether RA is required to limit anterior endoderm boundaries, we inhibited RA signaling with AGN193109 at stage HH 4. We found that *Hex* expression was unaffected (Table 2, not shown). Earlier inhibition (stage HH 3+) of RA signaling, however, laterally reduces *Hex* expression at HH 4 (Fig. 3C), whereas leaving these embryos longer until 15 somite stage results in ectopic *Hex* expression in foregut endoderm between the liver bud and thyroid gland (Fig. 3F; Table 2). This ectopic domain does not assume a complete thyroid or liver program as it does neither express *Nkx2.1* nor *Prox1* or *γ-fibrinogen* (Fig. 3I, L, O; Table 2, Fig. S3). Thus, inhibiting RAR activity in endodermal cells of the foregut reveals their competence to express *Hex* and we conclude that RA signaling is required until HH 3+ to restrict *Hex* expression to its endogenous domains.

We next analyzed *HoxB4*, which is endogenously expressed in all three germ layers including endoderm. By stage HH 13, *HoxB4* has reached its definitive anterior boundary in endoderm posterior to branchial arch 4 and just anterior to the first somite level (Fig. 3P). Increased RA signaling at gastrula and somite stages induced anterior shifts and elevated expression of *HoxB4* in endoderm, neural tube and surface ectoderm, which was confirmed by sectioning (n = 8; Fig. 3Q, T; Table 1). Similarly, *HoxA2*, which is endogenously expressed in all three germ layers including endoderm with an anterior boundary between branchial arches 2 and 3 [47], was anteriorly shifted by RA exposure at somite stages in ectoderm and endoderm (Fig. S2 A, B; Table 1). In the reverse experiment where we inhibited the RA signaling pathway with AGN193109 at HH 4 we found posteriorly shifted and decreased *HoxB4* mRNA levels (Fig. 3R; Table 2). However no change was detected in *HoxA2* expression pattern, possibly due to an earlier RA independence (Fig. S2 A, C; Table 2). Taken together these data show that RA signaling inhibits anterior branchial arch marker expression and that a defined level of activity positions the anterior boundary of branchial arch markers.

### RA is required to establish posterior foregut and midgut domains

**Figure 4:**
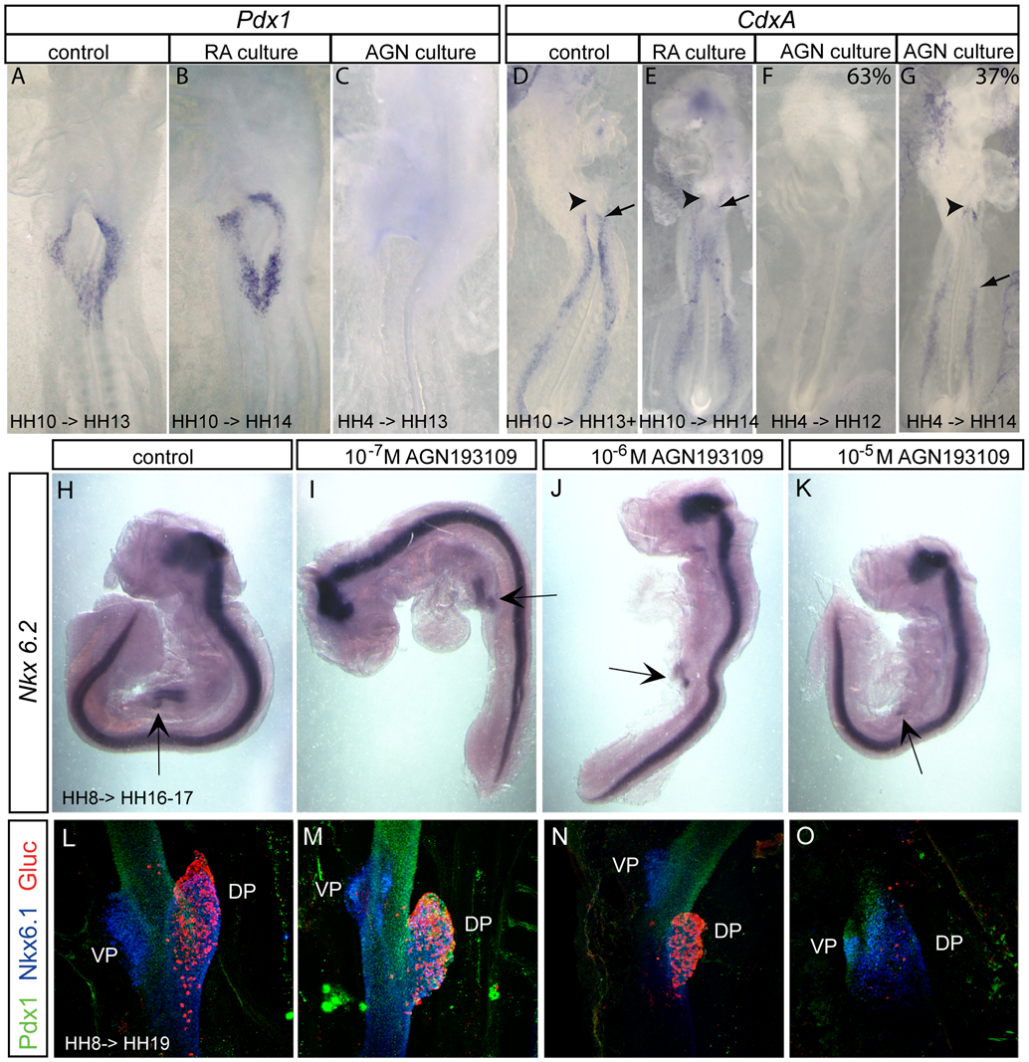
RA is essential to pattern the posterior foregut and midgut domains. Embryos are treated either with 10^−6^ M RA in the culture medium at HH 10 (B, E) or with 10^−5^ M AGN193109 in the culture medium at stage HH 4 (C,F,G) or HH 8 (D,E). Exact stages of treatment and analysis are indicated in each picture. Anterior is always to the top. Ventral view of whole mount *in situ* hybridized embryos for expression of *Pdx1* (A–C) and *CdxA* (D–G). RA activation had no effect on *Pdx1* or *CdxA* (B, E). Inhibition of RA resulted in complete inhibition of *Pdx1* (C) and two different phenotypes for *CdxA*. Either its expression was completely inhibited (F, 63%) or shifted towards posterior (G, 37%). Black arrowheads point to the AIP. Black arrows show the anterior boundary of *CdxA* expression. Note that the embryo in F has not formed the AIP properly. Side view of whole mount *in situ* hybridization for *Nkx6.2* after exposure to indicated amounts of AGN193109 in the culture medium from stage HH8 (4 somites) to HH16–17 (H–K). *Nkx6.2* expression in the pancreas is progressively reduced while expression in the nervous system is unaffected. Subsequent organogenesis was assessed at stage HH 19 by whole mount immunostaining for Pdx1 (pancreas and duodenum, green), Nkx6.1 (pancreas and subset of duodenum, blue) and glucagon (alpha cells, red) after exposure to indicated amounts of AGN193109 (L–O). Anterior is always to the top. (DP) Dorsal pancreas (VP) Ventral pancreas.

*Pdx1* is essential for pancreatic development and at HH 12, it marks a multipotent population of endoderm cells at the level of posterior foregut/anterior midgut, which give rise to caudal stomach, duodenum and dorsal and ventral pancreas. Embryos treated with RA at HH 4 or HH 10, either by bead grafting or applying RA to the culture medium, do not show any modification of *Pdx1* expression (Fig. 4A, B; Table 1).

*CdxA* is a gene expressed in endoderm that gives rise to the small intestine. At stage HH 13/14, we found its anterior boundary starts just caudally to the AIP (Fig. 4D) in a bilateral manner. Ventral and dorsal endoderm (liver primordium and dorsal pancreas primordia, respectively) are negative for *CdxA*. As development proceeds, *CdxA* domain progressively regresses and extends caudally. When we activated RA signaling at stage HH 4 or 10, *CdxA* gene expression was not anteriorly shifted (Fig. 4E; Table 1). Thus, RA alone is not sufficient to activate *Pdx1* and *CdxA* transcription outside of their endogenous expression domain.

By contrast, inhibition of RA signaling during gastrulation and early somitogenesis drastically prevents *Pdx1* expression (Fig. 4C; Table 2). The expression of the pancreas marker *Nkx6.2* is also blocked by AGN193109 in a dose dependent manner (Fig. 4H–K). This results in subsequent pancreas hypoplasia or in the extreme case an absence of pancreas (Fig. 4L–O). *CdxA* mRNA is either shifted posteriorly or completely inhibited (Fig. 4F, G, Table 2). Most embryos of the latter group show also defects in AIP closure. These results show that RA is needed for the expression of posterior foregut as well as midgut markers. Moreover, they demonstrate that disturbance of the patterning markers subsequently alters organogenesis.

### RA signals directly in endoderm to induce *CdxA* expression and pancreas formation

**Figure 5:**
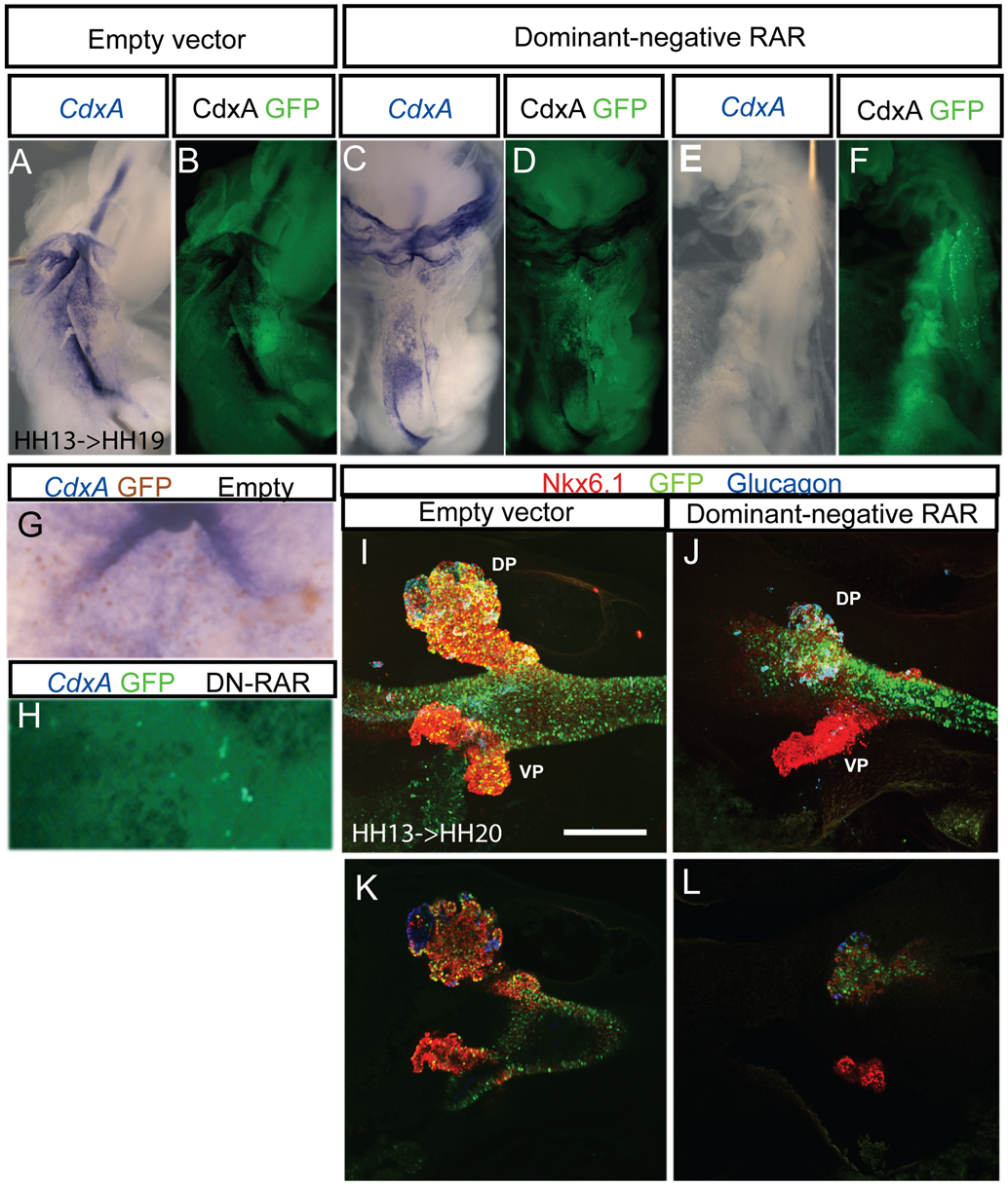
Electroporation of dominant negative RARs abolishes *CdxA* expression and pancreas formation. Electroporation of pCIG (A,B,G,I,K) or pCIG-DNRAR (C–F,H,J,L). Whole mount *in situ* hybridization on stage HH 19 embryos shows *CdxA* expression (blue) in the closed duodenum and the open midgut. *CdxA* expression is repressed either partially (n = 7/11, C) or completely (n = 3/11, F) by DN-RAR. Cells expressing the expression construct are labeled by subsequent immunocytochemistry for GFP expressed from the bicistronic construct (Green in D, F and H, masked by blue *CdxA* staining in B, DAB-brown in G), demonstrating that repression extends to the neighbors of targeted cells. Whole mount immunocytochemistry on stage HH 20 embryos shows that dominant negative RAR (traced with GFP, green) represses pancreas progenitor emergence (traced with Nkx6.1, red) in a non-cell autonomous manner (J) as compared to control embryos electroporated with empty vector (I). Glucagon+ cells could still differentiate (blue). (K and L) Selected optical sections of embryos displayed in (I) and (J), respectively. Scale bar 200 µm.

The expression of retinoic acid receptors and reporter genes for pathway activity such as *Cyp26A1* suggest direct activity in endoderm. We directly addressed the question by electroporating dominant negative retinoic acid receptors in endoderm. We observed that *CdxA* expression was either abolished (n = 3/11, highly electroporated embryos, not shown) or down-regulated (n = 7/11, lowly electroporated embryos) in endodermal cells expressing dominant negative receptors as compared to embryos electroporated with control plasmids (n = 16) (Fig. 5A–H). These results show that RA signaling is required directly in endoderm for *CdxA* expression. Moreover, *CdxA* expression was also repressed in neighboring endodermal cells several cell diameters away from the cells expressing dominant negative retinoic acid receptors (Fig. 5H). This shows that signaling between *CdxA* expressing cells is normally needed to maintain its expression.

Similarly, dominant negative RAR electroporation in endoderm led to an absence of the pancreas progenitor marker Nkx6.1 (n = 4) and reduction in pancreas size (Fig. 5I–L) although Pdx1 protein (n = 3) and RNA (n = 5) were unaffected (data not shown). Although chemical inhibition of pancreas formation abolishes glucagon-cell formation (Fig. 4L–O), numerous glucagon-positive cells were observed, possibly due to the late stage of electroporation. Accordingly, they were not GFP+. The requirement for RA in the ventral pancreas could not be addressed due to the inefficient targeting of ventral pancreas. These results show that direct RA signaling to endoderm is required in posterior foregut and midgut specification.

### RA acts synergistically with FGF4 in endoderm patterning rather than mediating its activity

Earlier studies in the nervous system have shown that both RA and FGFs induce posterior nervous system [58]. FGF4 has been shown to posteriorize endoderm in chick embryos during the same developmental period as RA [12]. The effects of RA and FGF4 on *Pdx1* and *CdxA* are different making it unlikely that one pathway mediates the activity of the other. Indeed, although both pathways are required for *Pdx1* and *CdxA* expression, only FGF4 induces anterior shifts of expression of these two markers. However, both FGF4 and RA block *Hex* expression anteriorly at gastrulation. To clarify if one pathway mediates the signaling of the other, we blocked one pathway and activated the other at stage HH 3+.

**Figure 6:**
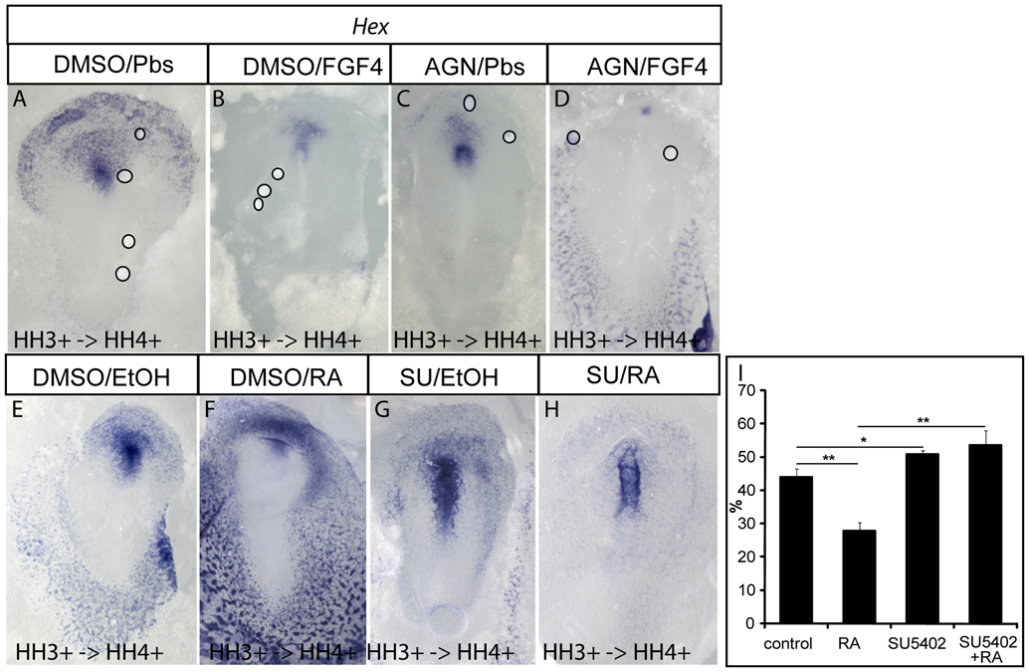
RA and FGF4 independently pattern the anterior endoderm. Whole mount *in situ* hybridization analysis of *Hex* expression. Ventral view, anterior to the top. (A–D) Analysis of embryos when FGF4 signaling is activated and RA signaling is inhibited. Embryos were treated at stage HH 3^+^ with DMSO and grafted with PBS beads (A) as control, treated with DMSO and grafted with FGF4 beads (1 mg/ml) (B), treated with10^−5^ M AGN193109 and grafted with PBS beads (C), or treated with 10^−5^ M AGN193109 and grafted with FGF4 beads (1 mg/ml) (D). Circles show position of beads in (A–D). (E–H) Analysis of embryos when FGF4 signaling is inhibited and RA signaling is activated. Embryos were treated at stage HH 3^+^ either with DMSO and ethanol (E) as control, with DMSO and 10^−6^ M RA (F), with 20 µM SU5402 and ethanol (G), or with 10^−6^ M RA and 20 µM SU5402 (H).Treatment was done at stage HH3^+^ and 24 embryos were analyzed 6 hours later at stage HH 4–5. Exact stages of treatment and analysis are indicated in each picture. (I) FGF4 activity is independent of RA. Embryos were treated and analyzed as (E–H). The “length of *Hex* domain/length of embryo” ratio in % was calculated (DMSO/ethanol n = 10, DMSO/RA n = 8, SU5402/ethanol n = 9, SU5402/RA n = 6). Bars in the diagram represent the mean and error bars display the standard error of the mean. The P-value was less than 0.001 (Student *t* test) between control and RA treated embryos and between RA treated and RA/SU5402 treated embryos (two asterisks). The P-value was less than 0.05 (Student *t* test) between control and SU5402 treated embryos (one asterisk). RA/SU5402 showed no significant difference from control embryos.

First, we activated FGF signaling by grafting heparin beads loaded with FGF4 (1 mg/ml) onto the ventral side of the embryos and inhibited RA signaling by including 10^−5^ M AGN193109 in the culture medium. FGF4 beads alone repressed *Hex* expression (4/7; Fig. 6B) as previously published [12]. As described above, the RA inhibitor alone caused lateral repression of the *Hex* domain (3/3, Fig. 3C and Fig. 6C). Activating the FGF signaling while blocking the RA pathway resulted in reduced *Hex* expression as when FGF4 was activated alone (4/8; Fig. 6D). This result shows that RA signaling is not needed downstream of FGF4 to repress *Hex*.

Then, we inhibited tyrosinase kinase activity of FGFR1 using SU5402 (20 µM) and activated RA signaling by adding RA (10^−6^ M), both into the culture medium. There was a high variation of *Hex* expression between similarly treated embryos. Therefore, we measured the length of the anterior *Hex* domain at the level of the midline and normalized to the total length of the embryo. By doing so, we considered only the expansion of the *Hex* domain along the AP axis, but we ignored possible phenotypes in lateral regions of the *Hex* expression domain. RA exposure alone resulted in reduced *Hex* expression in its anterior endodermal domain (*t*-test *P*<0,001; n = 8; Fig. 6F, I).

This repression was lost in the presence of SU5402 (n = 6; Fig. 6H, I), suggesting that *Hex* repression requires FGF signaling either downstream of or in parallel to RA signaling.

## Discussion

### RA signaling is required to establish gut tube domains along the main axis of the gut

**Figure 7:**
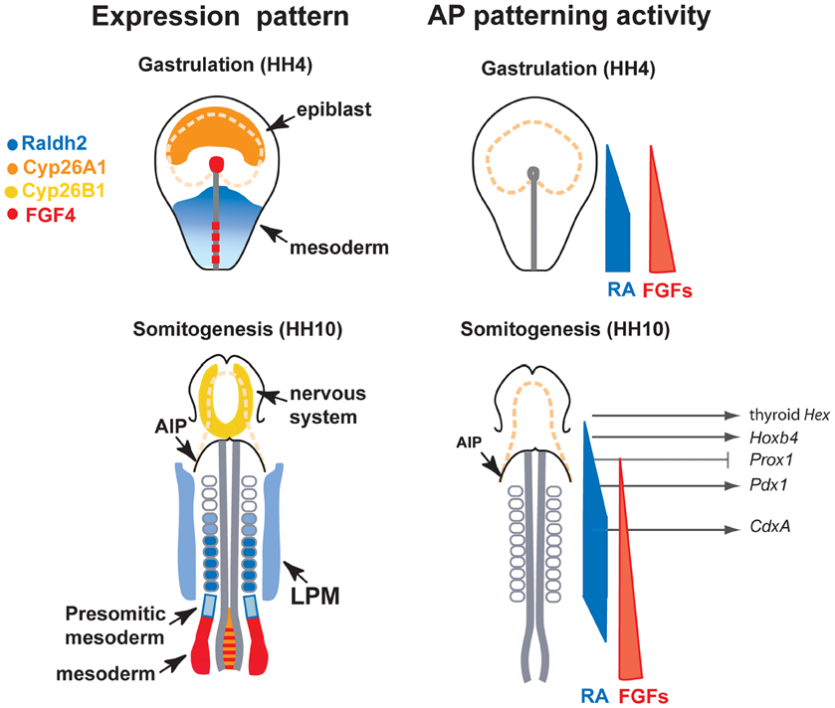
Model for AP patterning of RA and FGFs in endoderm. At gastrulation and early somitogenesis RA is synthesized in the posterior part of the embryo with its anterior limit around the junction between prospective fore- and hindgut. RA degrading enzyme *Cyp26A1* is expressed anteriorly and functions as intracellular sink for RA molecules (upper panel to the left). A RA gradient may be formed in the intermediate region. At the same time FGFs are expressed in the node and in the posterior streak acting in a graded manner along the entire AP axis (upper panel to the right). At stage HH 10 graded activation of RA signaling may be maintained in the dorsal (axial) endoderm anterior to the 6^th^ somite and in the foregut, while LPE is constantly exposured to RA (lower panel). FGFs are produced caudally in the tail bud (lower panel to the left), again acting in a graded manner along the AP axis. Posteriorly, RA may form a contra-gradient to FGFs antagonizing each other as it was shown in pre-somitic mesoderm. Different levels of signaling gradients induce different gene transcription. Whether FGFs also induce genes at the level of the posterior BAs, is not known (lower panel to the right). Color code is explained in the legend beside. Dotted orange lines represent presumptive neural plate. LPE, lateral plate endoderm; LPM, lateral plate mesoderm.

Although the roles of RA in a subset of the organ primordia investigated here have been reported in different species, our study uniquely provides a general overview of its activity coordinating the position of different endoderm organs along the AP axis, as schematized in Fig. 7. In spite of being largely consistent with previous observations in other species, we uncover important differences in the timing of RA activity as compared to non-Amniotes. Moreover, our work shows that RA is generally needed to generate all endoderm organs posterior to the branchial arches rather than a few.

### The thyroid forms in the absence or at very low levels of RA

Upon exposure to exogenous RA, we show that the thyroid markers *Hex* and *Nkx2.1* are repressed. *Hex* expression is required for *Nkx2.1* expression, which is essential for thyroid development [59]. In RA-treated *Xenopus laevis* or zebrafish, *Hex* expression in the thyroid was lost similarly to our results in chick embryos. Thus, increased RA prevents the specification of most-anterior endoderm to thyroid-fate, which is determined in the absence of, or at very low levels of RA (Fig. 7).

Although the effect of RA inhibition on thyroid development has been analyzed in several species, the results are somewhat controversial. *Hex* expression is expanded between the liver and thyroid in our stage 15 embryos when RA signaling is lost before HH 3+. This expansion does not correspond to liver or thyroid identity as neither *Nkx2.1*, *Prox* and *γ-fibrinogen* are expressed in this domain. Many signals are necessary to induce the liver and they may be missing in the *Hex* ^+^ domain between the liver and thyroid. In contrast, Stafford et al. observed a loss of thyroid *Hex* expression in vitamin A deficient quail (VAD) embryos [19]. Using VAD mimics loss-of-function of RA from the onset of embryogenesis, whereas we started inhibition of RA during early gastrulation. RA may, therefore, be required before gastrulation for later thyroid formation. Alternatively, the thyroid may form at very low concentrations of RA. RA inhibition in the VAD model may be more severe. Inhibitor treated embryos (HH 3+) analyzed earlier at HH 5 revealed, similarly as in VAD embryos [60], laterally reduced *Hex* expression. In HH 5 VAD embryos, expression of the Wnt inhibitor *Cres* is downregulated in lateral and anterior-most definitive endoderm resembling *Hex* expression in our RA inhibitor treated gastrula embryos [60]. Moreover, *Hex* is negatively regulated by the Wnt/β-catenin pathway in the hindgut of gastrulating *Xenopus laevis* embryos [13]. Therefore, we speculate that ectopically activated Wnt/β-catenin signaling inhibits *Hex* expression laterally in the absence of RA signaling. The effect of loss of RA signalling on *Hex* expression in the thyroid has been studied in three other models with different observations. In inhibitor-treated *Xenopus laevis* embryos, *Hex* expression was not expanded [18]. Such expansion may be difficult to see because in frogs liver and thyroid expressing *Hex* lie quite closely together. In *Raldh2* mutant zebrafish embryos, thyroid *Nkx2.1* was shifted posteriorly [17]. The posterior shift observed in zebrafish is arguable as the otic vesicle is used as a landmark and may have been itself shifted. Consistent with our experiments, thyroid development in mice does not require RA signaling [14]–[16], [61] but *Hex* expression was not specifically investigated in RA signaling-deficient mice.

### Graded levels of RA positions organs in branchial arches

Our observations of graded RA signaling activity in branchial arches is largely confirmatory of reports in other species. When we inhibited RA signaling at gastrulation stage, *HoxB4* was shifted posteriorly in chick endoderm. Loss of RA activity results in enlarged 2^nd^ pharyngeal arch (PA) and loss of 3^rd^ and 4^th^ PAs in mice [14]–[16], [61]. *HoxA1* and *HoxB1* are reduced in anterior endoderm of these mice [14], [16]. In our experiments *HoxA2* was not affected by RA loss possibly because it requires extremely low levels of RA or requires RA prior to stage HH3+.

Conversely, RA gain-of-function in our experiments shifts *HoxB4* and *HoxA2* expression anteriorly in endoderm suggesting that pharyngeal endoderm is abnormally posteriorized in mutant embryos. In *Crkl-* and *Tbx1-*deficient mice, ectopically activated RA signaling pathway correlates with ectopic anterior expression of *Hox* genes as well [61]. In amphioxus, *AmphiHox1* has been shown to mediate the effects of RA signaling by repressing expression of pharyngeal markers in the posterior foregut/midgut endoderm [62]. Our results obtained from chick embryos corroborate that RA is acting in a graded manner to pattern the foregut at the level of branchial arches. *Raldh2*, an enzyme responsible for RA production has a sharp anterior boundary in mesoderm at the level of the posterior foregut. RA activity in *RARE-lacZ* transgenics reaches more anterior endoderm areas up to the level of branchial arch 2, leading to the assumption that RA forms a diffusion gradient in branchial arches (Fig. 7). Moreover, expression of the reporter seems to be graded in the foregut [14], [21].

### RA is needed for pancreas formation

Our observations using chemical inhibition and direct inhibition in endoderm using dominant negative RARs reveal a requirement for RA signaling in early development of the dorsal pancreas in avian embryos as monitored by expression of *Pdx1*, *Nkx6.2*, Nkx6.1 (pancreas progenitors) and glucagon (differentiated endocrine cells). The requirement of RA for dorsal pancreas formation was also observed in other organisms including *Xenopus laevis*, zebrafish and mice [17]–[21]. In our experiments the entire *Pdx1*-expressing domain, which encompasses the dorsal and ventral pancreas as well as the duodenum, disappears. The effect on the ventral pancreas is less prominent in mice and frogs [18]. In contrast to observations in *Xenopus laevis* and zebrafish, we find that RA signalling is needed beyond gastrulation for proper pancreas development in the chick, at least until the onset of somitogenesis. In agreement with this late requirement, a complete rescue of *Pdx1* expression and endocrine pancreas differentiation in *Raldh2^−/−^* mice required RA treatment until E 9.5 [20], [21]. Moreover, it was recently shown by dnRAR-mediated inhibition of RA signaling in Pdx1-expressing cells in mice that RA is needed not only early, but also after pancreas specification to maintain dorsal and ventral pancreas progenitors [24]. This is confirmed in our electroporation experiments where RA inhibition occurs after the onset of *Pdx1* expression. Sequential expression of *Raldh2*, from mesoderm and *Raldh1* from pancreatic epithelium appear to provide RA [24].

In zebrafish and frogs, ectopic RA signaling resulted in anterior expansion of *Pdx1* [17]–[19]. This discrepancy may be correlated to the different timing requirements in Amniotes, to species-specific movements of the developing embryo, especially shifts between signaling mesoderm and endoderm. The fact that FGF4 can expand *Pdx1* more anteriorly [12] suggests that there is enough RA anterior to the pancreas area for *Pdx1* to be expressed (unless FGF4 induces RA signaling). Low levels of RA signaling anterior to the pancreas are therefore unlikely to define its anterior boundary. Moreover, reducing or increasing RA signaling, respectively, does not shift *Pdx1* posteriorly or anteriorly. Thus, a gradient of RA activity in posterior endoderm may exist, but *Pdx1* does not respond to this gradient.

### RA is directly needed in endoderm for *CdxA* expression

Our data on chick shows by two independent methods that RA signaling is required for *CdxA* expression. *Cdx* genes encode homeodomain transcription factors, and have been implicated as direct regulators of *Hox* expression in the nervous system [63]. In contrast to our observations, endodermal expression of the *CdxA* homologue *Zf-cad1* in zebrafish is not altered in response to either RA signaling or RAR inhibition [17]. Experiments in other species will be crucial to investigate the evolutionary conservation of this regulation. It is interesting to note that in the mouse mesoderm and ectoderm *Cdx1* expression also requires RA [64]–[67].

Our experiments also prove that direct RA signaling in endoderm is needed for *CdxA* expression. The non-cell autonomous effect suggests that endodermal cells with inappropriate RA signaling levels signal to their neighbors either directly or via the mesoderm to repress *CdxA*. It is unclear whether RA-low cells lack a positive signal needed to synchronize the cells in the *CdxA* field or send a negative signal.

### RA is not essential for initial liver induction but required for organogenesis

In our experiments, loss of RA does mildly affect liver formation. Liver development in *Raldh2* mutant mice, is not affected, since *Hex* and *Prox1* expression are still present in the ventral endoderm, where the liver forms [20], [21]. Likewise, liver *Hex* in *Xenopus laevis* and VAD embryos is not disturbed in the absence of RA signaling. In contrast in zebrafish, two late liver markers were inhibited when RA signaling was lost and *Hex* was partially lost. Using RA-soaked beads, *Prox1* but not *Hex* or *γ-fibrinogen* are locally inhibited. In *Hex^−/−^* embryos, *Prox1* is maintained [68]. From this, we conclude that RA signaling is not required to define the position of the future liver but rather interferes with liver maintenance or differentiation program.

### RA signaling is required for different periods of time depending on the AP level

The competence window during which cells activate gene expression upon RA treatment lasts at least from pre-gastrula until 10 somite stage. However, manipulation of chick embryos with the RAR blocker AGN193109 demonstrates that the response to endogenous RA signaling is lost gradually, with anterior structures becoming independent from RA signaling before posterior structures. Accordingly, *Hex* expression in the thyroid can be changed only prior to stage HH 3+. *HoxB4* expression, which is positioned more posterior to *Hex,* can be still shifted posteriorly at stage HH 4 but not at stage HH 10. Of interest, Wendling et al observed a narrow developmental time window between 7–10 somite stage (around E 8.25) in which absence of RA only could affect the formation of 3^rd^ and 4^th^ PA structure in mice [14]. This suggests that the time window at which stage which level along the AP axis can be affected slightly differs between Amniotes. *Pdx1* expression in the posterior foregut is inhibited at 4 somite stage when RA signaling is impaired, while later manipulations (HH 10) do not affect *Pdx1* mRNA expression. Lastly, the posterior-most marker we used, *CdxA,* still shows a posterior shift in a subset of embryos when RA signaling is blocked by applying the inhibitor as late as stage HH 10 (Table 2) or upon dominant negative RAR electroporation at stage HH 13–14. Therefore, the more posterior an endodermal cell is located, the longer RA signaling is required. Posterior cells may acquire more posterior identities as a consequence of longer RA exposure posteriorly (duration) or later RA exposure (competency-delay of posterior development). Our results also show that RA is needed beyond gastrulation in Amniotes. The mode of gut tube lumen formation is different between non-Amniotes and Amniotes. In the later, folding brings cells that are initially anteriorly located to more posterior positions in the ventral part of the gut [69]. Signaling may be needed until they reach their final position to keep in register the AP identity of dorsal and ventral cells, some of which will contribute to the same organs.

We also provide some arguments in favor of a physical RA gradient: Between HH 5 and HH 7, *Raldh2* is expressed in a fan-shape in the posterior half of chick embryos with its anterior border at the 1^st^ somite and with a *Raldh2*-low region laterally (Fig. 1A and not shown). RA is presumed to diffuse through tissues [69], therefore, capable to enter RA non-synthesizing areas. The stable anterior expression boundary of *Raldh2* argues for a graded RA exposure of endoderm in the *Raldh2*-negative anterior region and the lateral *Raldh2*-low area from which part of the midgut/hindgut cells originate (Fig. 6). RA is then degraded intracellularly in the anterior region of the embryo by Cyp26 enzymes allowing the expression of genes that are normally repressed by RA [70]. A second observation in favor of a gradient is that *HoxB4* expression is repressed more efficiently in its BA domain than in midgut upon RAR inhibitor treatment suggesting that higher doses of RA posteriorly may not be completely inhibited. Similarly, *CdxA* is repressed more efficiently in its anterior domain upon RA inhibition. At early somitogenesis, *Raldh2* expression argues for a gradient of RA synthesis in anterior somites. *Raldh2* shifts posteriorly with time. In agreement, *Raldh2* is detected at E 8.5 in all somites, whereas at E 9.5 expression in rostral somites became faint [71]. The *Raldh2* expression data provide evidence for a RA gradient in dorsal endoderm from 1 to 6 somite level, whereas LPE, which becomes lateral and ventral gut tube, is evenly exposed to RA (Fig. 6) Taken together, endoderm patterning by RA may be the result of both duration of RA exposure and the concentration of signal at a given place in endoderm.

### Coordination between signaling pathways

We investigated possible interactions between FGF and RA signaling pathway in endoderm patterning during gastrulation where both pathways have been shown to be active [12].

Our results in endoderm show that the effect of the FGF and RA pathways are not mediated by one another. Firstly, RA and FGF4 have distinct effects on *Pdx1* and *CdxA* expression. Indeed FGF4 shifts expression of these genes anteriorly, whereas RA does not. Secondly, although they have the same effect on *Hex*, FGF4 mediated repression does not require RA signaling. However, RA signaling is not sufficient to block *Hex* in the absence of FGF signaling suggesting that these pathways block this gene in synergy or that FGF4 mediates RA activity.

RA has been used to promote the formation of pancreatic beta cells from ES cells [72]–[74]. Our experiments together with previous work argue that RA may be needed in the generation of more posterior endodermal cell types in vitro such as intestines and liver and on the contrary may be detrimental to the generation of thyroid cells. Together with previously published data, they show that RA is needed not only for the early induction of these organs but also for the maintenance of their progenitors, at least for the liver, pancreas and intestine.

## Supporting Information

Figure S1 Selection of RAR inhibitors. Two inhibitors of the RA pathway were tested in RA-responsive P19 embryonic carcinoma cells and assayed by PCR for activation of the RA pathway target *RARβ*. RA and the agonist AGN190121 activate *RARβ* at comparable levels [30]. AGN193109 efficiently blocks *RARβ* induction by RA or AGN190121 whereas BMS453, a RARβ agonist but RARα and RARγ antagonist [15], did not. TBP (TATA box binding protein, also called TFIID, GenBank acc. no. D01034) is used for normalization.
(3.84 MB TIF)

Figure S2 RA shifts *HoxA2* anteriorly. Embryos are treated either with 10^−3^ M RA loaded on beads at HH 10 (B,D) or with 10^−5^ M AGN193109 in the culture medium at stage HH 3^+^ (C). Control embryos are shown in (A). Anterior is always to the top. Whole mount *in situ* hybridized embryos for expression of *HoxA2* (A–D). RA shifts *HoxA2* anteriorly in the nervous system (upper arrow in B) and in the endoderm (lower arrow in B and arrows in section in D). RA inhibition did not change *HoxA2* expression pattern (C).
(11.96 MB TIF)

Figure S3 RA does not significantly modify *γ-fibrinogen* expression. Embryos are treated either with 10^−3^ M RA loaded on beads at HH 10 (B,E) or with 10^−5^ M AGN193109 in the culture medium at stage HH 3^+^ (C,F). Control embryos are shown in (A,D). Anterior is always to the top. Whole mount *in situ* hybridized embryos for expression of *γ-fibrinogen* (A–F) shows this marker in the liver and extraembryonic endoderm in control embryos (A, F, faint expression shown by arrow). A RA-soaked bead does not affect *γ-fibrinogen* expression (arrow in B,E). RA inhibition did only slightly but reproducibly up-regulate *γ-fibrinogen* expression (C,F).
(5.27 MB TIF)
