# Supplementary figures and images for: Retinoic Acid Signaling Organizes Endodermal Organ Specification along the Entire Antero-Posterior Axis
Source: PLoS One. 2009 Jun 10;4(6):e5845. doi: 10.1371/journal.pone.0005845 (PMC2690404; doi:10.1371/journal.pone.0005845)

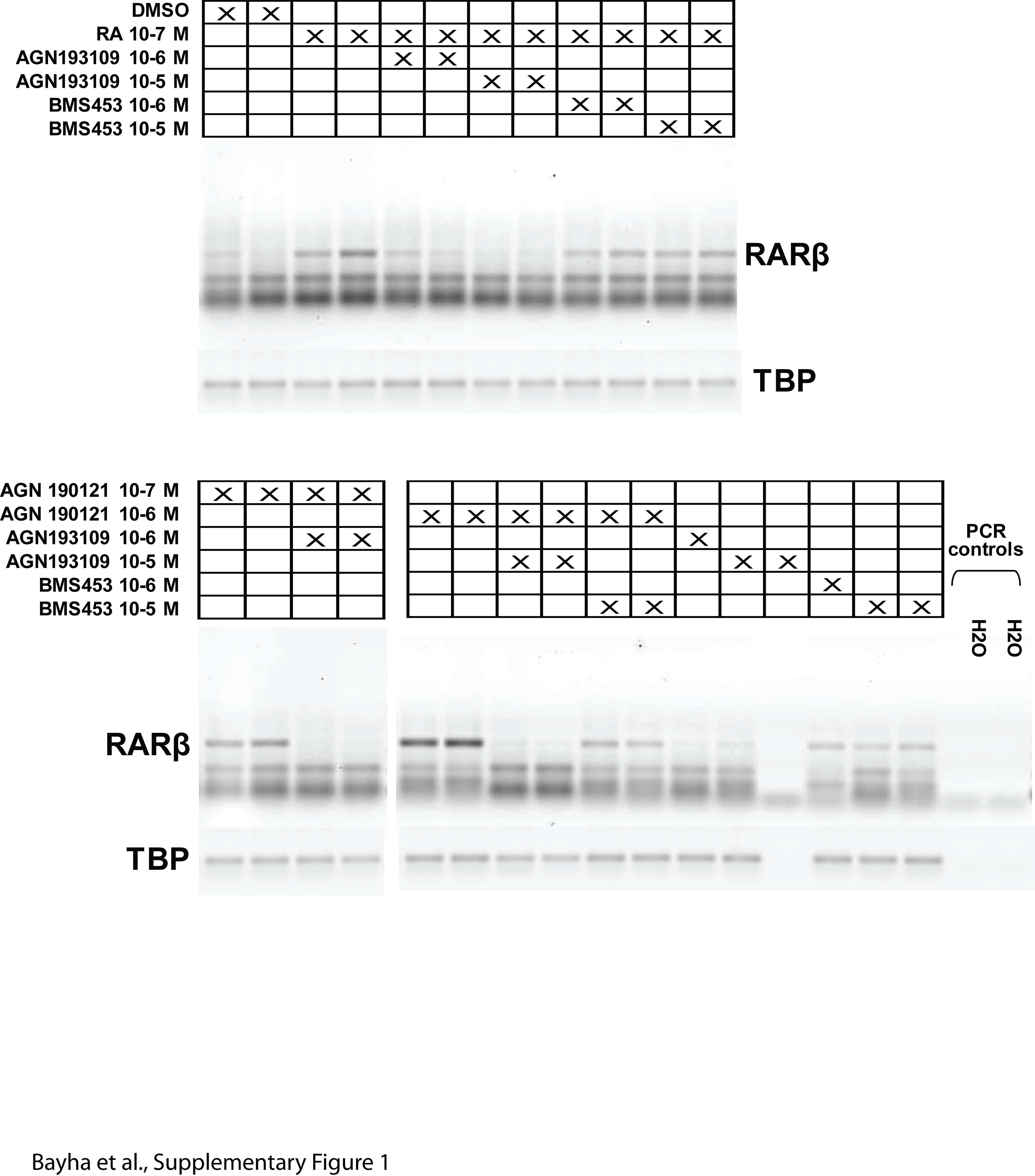

Supplement: Figure S1 — Selection of RAR inhibitors. Two inhibitors of the RA pathway were tested in RA-responsive P19 embryonic carcinoma cells and assayed by PCR for activation of the RA pathway target RARβ. RA and the agonist AGN190121 activate RARβ at comparable levels [30]. AGN193109 efficiently blocks RARβ induction by RA or AGN190121 whereas BMS453, a RARβ agonist but RARα and RARγ antagonist [15], did not. TBP (TATA box binding protein, also called TFIID, GenBank acc. no. D01034) is used for normalization. (3.84 MB TIF) [file pone.0005845.s001.tif]

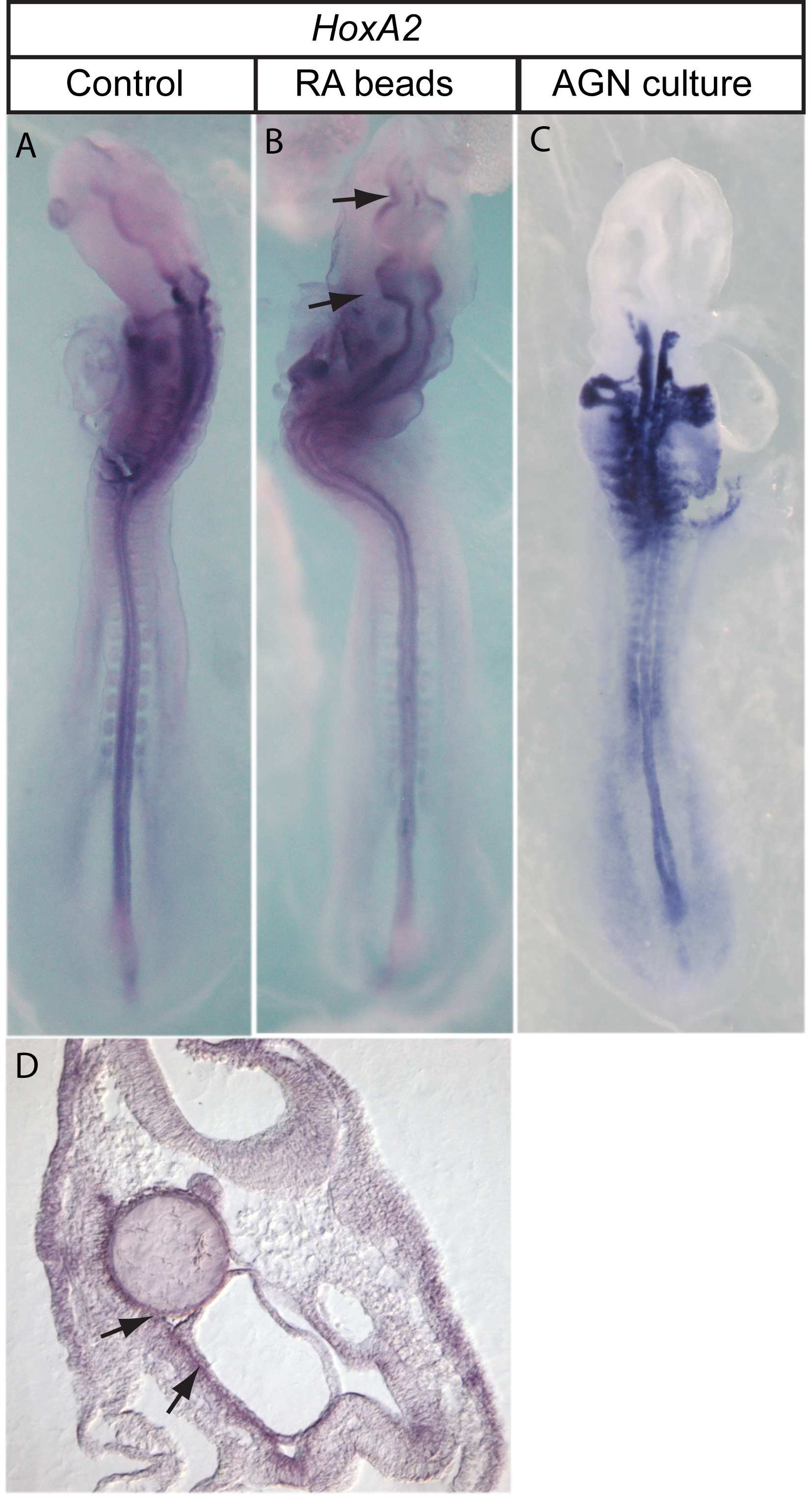

Supplement: Figure S2 — RA shifts HoxA2 anteriorly. Embryos are treated either with 10−3 M RA loaded on beads at HH 10 (B,D) or with 10−5 M AGN193109 in the culture medium at stage HH 3+ (C). Control embryos are shown in (A). Anterior is always to the top. Whole mount in situ hybridized embryos for expression of HoxA2 (A–D). RA shifts HoxA2 anteriorly in the nervous system (upper arrow in B) and in the endoderm (lower arrow in B and arrows in section in D). RA inhibition did not change HoxA2 expression pattern (C). (11.96 MB TIF) [file pone.0005845.s002.tif]

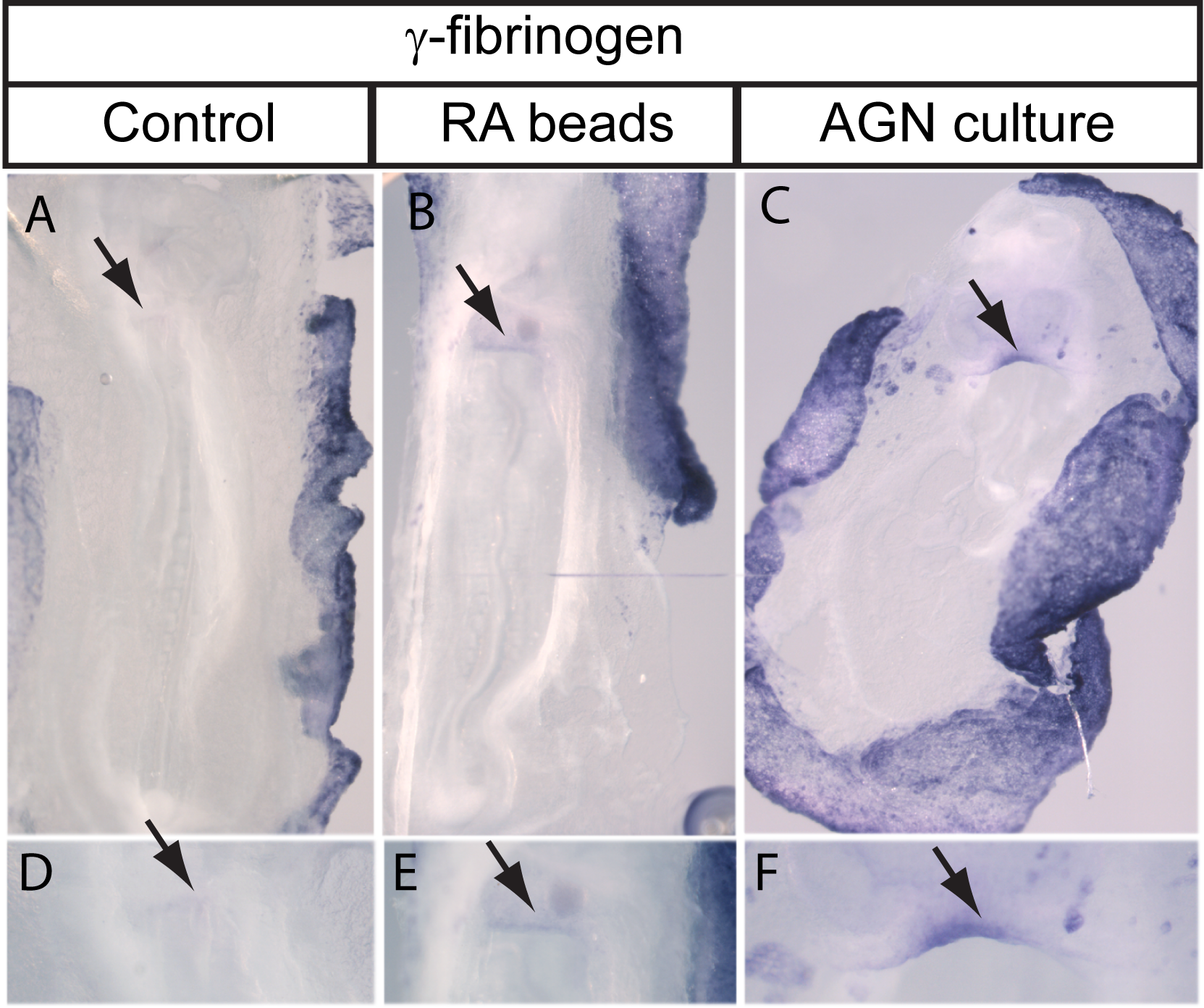

Supplement: Figure S3 — RA does not significantly modify γ-fibrinogen expression. Embryos are treated either with 10−3 M RA loaded on beads at HH 10 (B,E) or with 10−5 M AGN193109 in the culture medium at stage HH 3+ (C,F). Control embryos are shown in (A,D). Anterior is always to the top. Whole mount in situ hybridized embryos for expression of γ-fibrinogen (A–F) shows this marker in the liver and extraembryonic endoderm in control embryos (A, F, faint expression shown by arrow). A RA-soaked bead does not affect γ-fibrinogen expression (arrow in B,E). RA inhibition did only slightly but reproducibly up-regulate γ-fibrinogen expression (C,F). (5.27 MB TIF) [file pone.0005845.s003.tif]
